# Atypical Presentation of Antenatal Eclampsia

**DOI:** 10.7759/cureus.24745

**Published:** 2022-05-05

**Authors:** Mohan V. Sumedha Maturu, Shanthi Pappu, Aravind Varma Datla, Anil Devara, Sibasankar Dalai

**Affiliations:** 1 Neurology, Medicover Hospitals, Visakhapatnam, IND; 2 Obstetrics and Gynecology, Medicover Hospitals, Visakhapatnam, IND; 3 Internal Medicine, Medicover Hospitals, Visakhapatnam, IND; 4 Interventional Radiology, Medicover Hospitals, Visakhapatnam, IND; 5 Interventional Neuroradiology, Medicover Hospitals, Visakhapatnam, IND

**Keywords:** mri- magnetic resonance imaging, bilateral blurring of vision, generalized tonic-clonic seizures, cesarean section (cs), posterior reversible encephalopathy syndrome (pres), proteinuria, preeclampsia-eclampsia, normotensive eclampsia, hypertensive disorders of pregnancy, atypical eclampsia

## Abstract

Most women who develop eclampsia have preceding preeclampsia (proteinuria and hypertension). This is especially true for otherwise healthy nulliparous women. However, recently, there has been a paradigm shift in this philosophy. There is mounting evidence that preeclampsia can develop even in the absence of proteinuria and hypertension and that eclampsia itself may be the initial manifestation of hypertensive disorder during pregnancy. We report a rare case of a 24-year-old primigravida at 30 weeks of gestation who presented with new-onset generalised tonic-clonic seizures without prior hypertension or proteinuria in her antenatal records. A thorough workup revealed this presentation to be the initial feature of atypical eclampsia. She was managed appropriately and discharged with an excellent outcome. This experience highlights some of the difficulties in managing a case of atypical eclampsia, namely, erratic onset and an unpredictable course, all of which interfere with timely diagnosis and treatment and contribute to maternal and fetal morbidity and mortality.

## Introduction

Eclampsia is the new onset of generalised tonic-clonic seizures or coma in a patient with the preeclamptic triad of oedema, hypertension, and proteinuria [1]. However, its exact etiopathogenesis is poorly understood. Eclampsia is a neurological complication of pregnancy and significantly contributes to maternal morbidity and mortality [2]. Eclampsia is a diagnosis of exclusion. Other underlying medical and neurological disorders that can cause these signs and symptoms should be ruled out. Oedema is no longer a critical criterion because it is a common observation in a typical pregnancy, and about one-third of eclamptic women do not develop oedema [3].

The term "atypical preeclampsia-eclampsia" is being increasingly used to represent aberrant forms of hypertensive disorders arising during pregnancy [3,4]. Atypical preeclampsia-eclampsia lacks a categorical definition and can include no or minimal proteinuria with hypertension, no or minimal blood pressure elevation with proteinuria, or absence of both proteinuria and hypertension. Other presentations that can be included in this blanket atypical category are onset before 20 weeks gestation or after 48 hrs postpartum, magnesium sulphate therapy resistance, hemolytic anaemia with elevated liver enzymes, and low platelets (HELLP) syndrome and its variants such as elevated liver enzymes-low platelets (ELLP) and elevated liver enzymes (EL) [3,5]. Atypical eclampsia accounts for roughly 8% of all eclampsia cases. Atypical forms of eclampsia have erratic onsets. The process of making the correct diagnosis and initiating timely therapy is arduous. These factors contribute to morbidity and mortality [3].

To highlight some of the challenges in managing such scenarios, we present a case of a young primigravida patient who presented with new-onset generalised seizures in the background of normal antenatal records. Based on the presentation and subsequent investigations, a diagnosis of atypical eclampsia was made, and the patient was managed accordingly, resulting in the avoidance of any devastating permanent sequelae and a good overall outcome.

## Case presentation

In January 2022, during the peak of the third wave of Coronavirus disease (COVID-19) in India, a 24-year-old primigravida presented to the emergency department in the late evening at 30 weeks of gestation with one episode of generalised seizures one hour back. At the presentation, she was conscious, coherent and cooperative. Her vitals were: blood pressure 110/70 mmHg, pulse rate 88 beats per minute, respiratory rate 27 breaths per minute, temperature 98.7°F, and oxygen saturation 97% on room air. Examination of the cardiovascular and respiratory systems was normal. There were no focal neurological deficits. Abdominal and pelvic examination revealed a uterus height corresponding to 30 weeks of gestation, cephalic presentation of the fetus, fetal heart rate 140 per minute, relaxed uterus, uneffaced cervix, and a closed os.

Within 30 minutes of the presentation, while still in the emergency department, the patient had a second episode of generalised tonic-clonic seizures. She was given a 2 mg bolus of intravenous (IV) midazolam which terminated the seizure. Her antenatal records revealed normal blood pressures and no evidence of proteinuria. She also received a loading dose of IV levetiracetam, and a magnetic resonance imaging (MRI) of the brain with magnetic resonance - time of flight (MR - TOF) venogram was attempted, but given post-ictal delirium, there was limited patient co-operation. However, with the limited clarity, we could decipher only the diffusion-weighted imaging (DWI) sequence, which showed no abnormality. A repeat MRI brain was planned once she recovered from the delirium. Initial possibilities of acute viral encephalitis (because of the concomitant COVID wave), cerebral venous sinus thrombosis, and the rare possibility of atypical eclampsia were considered. She was also started empirically on ceftriaxone 2gm IV twice daily, IV acyclovir 15mg/kg/dose thrice daily. Enoxaparin 60mg s/c stat dose was given, and the continuation was planned as per the repeat MRI findings. IV magnesium sulphate (MgSO4) was initiated at a loading dose of 4gm over 20 minutes, followed by a maintenance dose of 2g per hour as a continuous IV infusion for the next 48 hours. Blood workup work for sepsis markers, electrolytes, renal and liver functions were normal. Urinary dipstick test showed 2+ proteinuria. She was monitored in the intensive care unit (ICU). She gradually regained her sensorium and slept for the night. 

**Figure 1:**
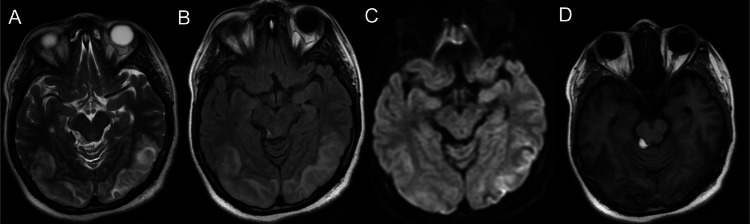
MRI at the level of the midbrain. The altered signal intensity which appears hyperintense on T2WI (A), FLAIR (B); T2 shine through in DWI (C); hypointense on T1WI (D) involving bilateral posterior temporal and temporo-occipital regions. An incidental midbrain lipoma of size 11.6 X 8mm is noted which is hyperintense on T1WI (D). DWI - Diffusion-weighted imaging FLAIR - Fluid attenuated inversion recovery MRI - Magnetic resonance imaging T1WI - T1 weighted imaging T2WI - T2 weighted imaging

**Figure 2:**
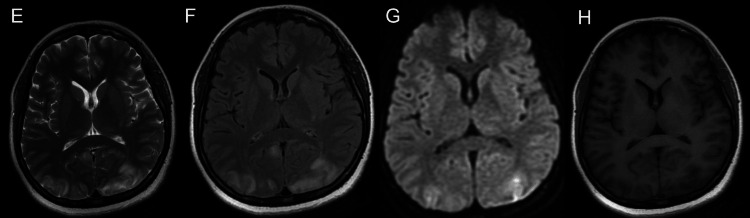
MRI at the level of internal capsule. Altered signal intensity which appears hyperintense on T2WI (E), FLAIR (F); T2 shine through in DWI (G); hypointense on T1WI (H) involving bilateral parieto-occipital regions. DWI - Diffusion-weighted imaging FLAIR - Fluid attenuated inversion recovery MRI - Magnetic resonance imaging T1WI - T1 weighted imaging T2WI - T2 weighted imaging

**Figure 3:**
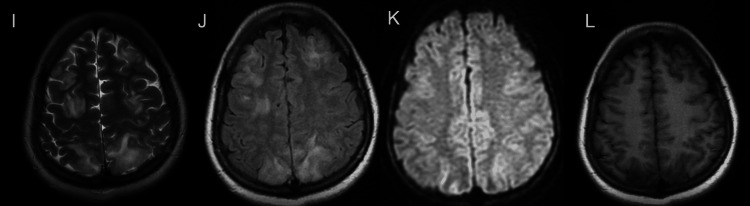
MRI at the level of centrum semiovale. Altered signal intensity which appears hyperintense on T2WI (I), FLAIR (J); T2 shine through in DWI (K); hypointense on T1WI (L) involving bilateral posterior parietal and high frontal regions. DWI - Diffusion-weighted imaging FLAIR - Fluid attenuated inversion recovery MRI - Magnetic resonance imaging T1WI - T1 weighted imaging T2WI - T2 weighted imaging

**Figure 4:**
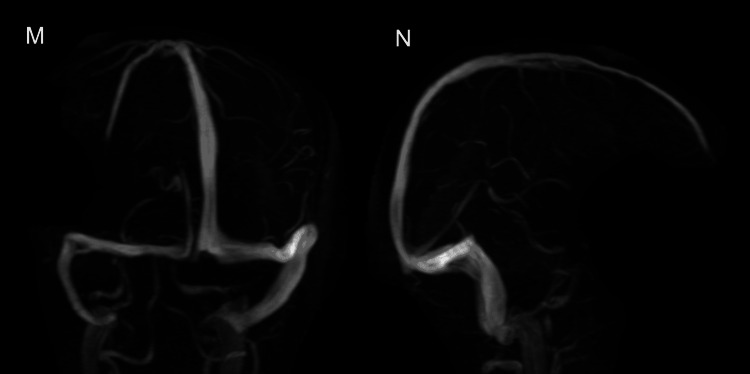
MR venogram – TOF sequence showing normal venous sinuses. Anteroposterior (M) and lateral (N) views MR - Magnetic resonance TOF - Time of flight

The following day, roughly 12 hours after the second seizure, she reported a sudden onset of blurred vision which rapidly progressed to a state where she only had the perception of light in both eyes. A repeat emergent MRI of the brain was performed (Figure 1-4), which showed T2-weighted-fluid-attenuated inversion recovery (T2/FLAIR) hyperintensities without corresponding diffusion-weighted imaging (DWI) restriction predominantly involving the white matter of the temporo-parieto-occipital region, which was highly suggestive of posterior reversible encephalopathy syndrome (PRES). Repeat blood pressure was checked after seeing the MRI findings and was normal. In light of these findings, the patient was classified as a case of atypical antenatal eclampsia without hypertension. 

The patient was shifted to the operation theatre, and an emergency lower (uterine) segment Caesarean section (LSCS) was performed under general anaesthesia. A live male child weighing 1.2 kg with an Apgar score of eight was delivered. Uterine atony resulted in postpartum haemorrhage (PPH), which was controlled with intramuscular carboprost. Postoperatively, she was managed with IV anti-oedema measures and IV anti-epileptic maintenance. Her vision gradually improved and was near-normal by 48 hours post-delivery. Anti-edema measures were gradually tapered, and she was discharged seven days post-delivery with normal vision. During the entire hospital stay, her blood pressure recordings were never high. At her three-month follow-up, she did not have any neurological complications. Anti-epileptics were tapered and stopped.

## Discussion

The incidence of eclampsia varies widely throughout the world. It ranges from 1.5 - 10 cases per 10,000 deliveries in high resource countries to 19.6 - 142 per 10,000 deliveries in the middle, low and exceedingly resource-poor countries [6].

Classically, eclampsia has been viewed as the final stage of a disease progression that starts subclinically and progresses to preeclampsia before ultimately winding up as eclampsia. This leads to the implication that proteinuria and hypertension precede the development of eclampsia. In sharp contrast to this philosophy, eclampsia can be encountered quite early before the onset of proteinuria and hypertension, which has led to the criticism that the term "preeclampsia" can be misleading [7]. The International Society for the Study of Hypertension in Pregnancy (ISSHP) has updated the guidelines for the classification, diagnosis, and management of hypertensive disorders of pregnancy (Table 1) [8].

**Table 1:** The 2018 ISSHP classification for hypertensive disorders of pregnancy. ISSHP -  International Society for the Study of Hypertension in Pregnancy

Hypertension known before pregnancy or present in the first 20 weeks
1. Chronic hypertension
a. Essential
b. Secondary
2. White-coat hypertension
3. Masked Hypertension
Hypertension arising de novo at or after 20 weeks
1. Transient gestational hypertension
2. Gestational hypertension
3. Pre-eclampsia – de novo or superimposed on chronic hypertension

In the classical forms of eclampsia, arterial and renal systems are the first to be involved, manifesting as hypertension and proteinuria before other organ systems manifestations [9]. However, in atypical eclampsia, cerebral involvement can precede other organ systems dysfunction [5]. 

In our case, cerebral involvement was the first feature. A high degree of suspicion is required to constantly monitor for organ malfunction in suspected cases without overt features of preeclampsia. In our patient, the diagnosis of atypical eclampsia was based on the previously mentioned clinical (seizures), biochemical (urine proteins) and radiological (MRI brain) features with the absence of other underlying pathologies (infections, autoimmune disorders, cerebral venous sinus thrombosis).

Our patient is an example where the natural progression of the disease process deviated from that of typical eclampsia. Firstly, a thorough review of her antenatal records showed no evidence of hypertension or proteinuria. Her previous visit was two-three weeks prior to the current admission. The sudden detection of proteinuria can be an abrupt affair, characteristic of atypical eclampsia or a post-ictal event [10]. However, features such as an acute visual disturbance and typical bilateral symmetrical hyperintensities on T2 weighted images involving the parietal and occipital lobes white matter changes without alternative explanations clinched the diagnosis. PRES is the most common neuroimaging finding in eclampsia. The co-incidence can be as high as 90-98% [11,12].

The initial priority in managing a case of eclampsia is to maintain airway patency and prevent aspiration. Supplemental oxygen may be given to combat maternal hypoventilation and hypoxemia during a seizure. Maternal trauma should be prevented. If present, hypertension should be promptly treated. Labetalol and hydralazine are preferred over nicardipine and nifedipine [13]. 

Magnesium sulphate (MgSO_4_) is the preferred antiseizure medication. It is primarily used to prevent recurrent seizures rather than control initial seizures. About 10% of patients tend to have recurrent seizures [14]. Recurrent seizures can lead to rhabdomyolysis, neuronal death, metabolic acidosis, neurogenic pulmonary oedema, aspiration pneumonitis, and respiratory failure. Multiple studies reported that magnesium sulphate is safer and more effective than diazepam, phenytoin, or lytic cocktail (i.e., promethazine, chlorpromazine, and pethidine). The likelihood of recurrent seizures decreases by 33 - 50% with MgSO_4_ compared to diazepam and phenytoin [15,16,17]. The advantages of MgSO_4_ over other antiseizure medications are its low cost, no need for cardiac monitoring, and no sedation. In addition, patients administered with MgSO_4_ are less likely to require ventilatory support, intensive care admission, and develop pneumonia. Moreover, in utero exposure to MgSO_4_ therapy decreases the risk of cerebral palsy and severe motor dysfunction in offspring born before 32 to 34 weeks of gestation due to its neuroprotective effects [15,16,18]. Hypomagnesemia is a common finding in PRES and a possible etiological factor. Hence, some have suggested that magnesium supplementation may be a helpful adjunct in PRES management and the drug of choice for seizure control in eclampsia. MgSO_4_ is also a potent cerebral vasodilator [19,20].

A typical administration regimen is a loading dose of 4 to 6 gm IV over 15 to 20 minutes, followed by a maintenance dose of 2gm per hour. Intramuscular administration has a slower onset of action. If seizures recur, a repeat bolus of 2 to 4gm can be given over five minutes. Refractory seizures may require other anti-epileptic measures and invasive mechanical ventilation. All the while, the constant vigilance of fetal activity is of paramount importance [3,13].

Prompt delivery is the definitive treatment for eclampsia. This is even more important if the fetal heart tracing does not improve 10 to 15 minutes after maternal stabilisation and fetal resuscitative measures [13,20]. Considering our patient's neurological, cervical status and that she was not in labour, an emergency caesarean section was performed. 

## Conclusions

The incidence of eclampsia continues to be high in middle and low-income countries. Eclampsia contributes to poorer maternal and fetal outcomes. This is especially true for atypical presentations. A high index of suspicion is needed to promptly recognise atypical eclampsia and to initiate timely therapy and termination of pregnancy.
